# Violence-related periorbital trauma with a retained foreign body: a case report

**DOI:** 10.1186/s13256-015-0779-1

**Published:** 2016-01-20

**Authors:** Giovanni Dell’Aversana, Gaetano Marenzi, Pasquale Piombino, Domenico Testa, Giacomo De Riu, Vincenzo Abbate, Luigi Califano, Gilberto Sammartino

**Affiliations:** 1 Division of Maxillofacial Surgery, Department of Neurosciences, Reproductive and Odontostomatological Sciences, University of Naples “Federico II”, Via Pansini, 5, 80131 Naples, Italy; 2 Oral Surgery Division, Department of Neurosciences, Reproductive and Odontostomatological Sciences, University of Naples “Federico II”, Via Pansini, 5, 80131 Naples, Italy; 3 Ear, Nose and Throat Department, Second University of Naples, Via Pansini, 5, 80131 Naples, Italy; 4 Maxillofacial Department, University of Sassari, Viale San Pietro, 43/B 07100, Sassari, Italy

**Keywords:** Orbital floor fracture, Computed tomography, Foreign body, Blunt trauma, Hemosinus

## Abstract

**Background:**

Orbital fracture usually occurs as a result of blunt orbital and facial trauma and may involve ocular injuries. International studies on orbital floor fracture show several differences in epidemiology, diagnostic criteria, surgical treatment modalities, and complication rates; therefore, any comparison should be made with caution. Here we describe an unusual case involving a 19-year-old man with violence-related periorbital trauma, wherein a foreign body (a plastic pen cap) traversed the median wall of the maxillary sinus and penetrated the lower turbinate.

**Case presentation:**

A 19-year-old Caucasian man was referred to our department with localized pain and swelling in the left suborbital region following a physical fight in May 2014. A clinical examination revealed no abnormalities in his eyeballs or eye movement, palpation of the orbital contour revealed no fractures, and ophthalmological evaluation showed no evidence of diplopia. A computed tomography scan revealed fractures in the left orbital floor, periorbital tissue herniation without muscular entrapment and left maxillary hemosinus were observed. A hypodense soft tissue mass was lodged in the left orbital floor, which extended to the median wall of the maxillary sinus and penetrated the left lower turbinate. Surgical exploration of the foreign body was conducted, revealing the foreign body to be a pen cap.

**Conclusions:**

History or clinical examination alone may be inadequate to raise the suspicion of a retained periorbital foreign body in a situation of orbital region trauma. Computed tomography is important for the evaluation of periorbital injuries, especially because it could reveal the presence of a foreign body. Periorbital foreign bodies can be observed distinctly on computed tomography, which remains the most sensitive study and should be the first imaging modality in such cases.

## Background

Orbital fracture usually occurs as a result of blunt orbital and facial trauma and may involve ocular injuries [1, 2]. Young adults and teenagers are predominantly affected [3], with motor vehicle accidents being the leading cause, followed by falls and sports injuries [1–4]. More recent studies have identified assault as the main cause [5]. In general, patients exhibit polytrauma and require multidisciplinary treatment involving various medical specialties such as ophthalmology, otorhinolaryngology, neurosurgery, and plastic surgery for the restoration of function and aesthetics. International studies on orbital floor fracture show several differences in epidemiology, diagnostic criteria, surgical treatment modalities, and complication rates; therefore, any comparison should be made with caution [6]. Here we describe an unusual case involving a 19-year-old man with violence-related periorbital trauma, wherein a foreign body (a plastic pen cap) traversed the median wall of the maxillary sinus and penetrated the lower turbinate. Our patient was hospitalized for evaluation and removal of the foreign body to prevent severe infection. This case is rare because it demonstrates how in such situations orbital trauma could be misdiagnosed or not properly approached due to incorrect evaluation.

## Case presentation

**Fig. 1:**
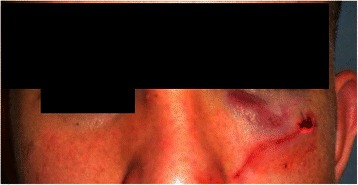
Clinical examination. Moderate edema and ecchymosis of the suborbital left region, bruising of the left lower eyelid, and skin sutures at the outer third of the orbital frame can be observed

**Fig. 2:**
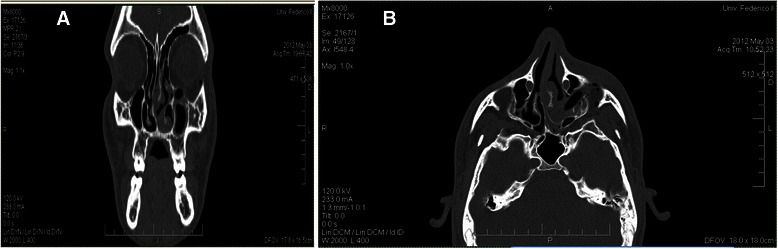
**a**–**b** Axial and coronal views of computed tomography images. A left orbital floor fracture and a foreign body localized in the medial portion of the left orbital floor, which extends upward to the left lower turbinate, can be observed

A 19-year-old Caucasian man was referred to the Department of Neurosciences, Reproductive and Odontostomatological Sciences, University of Naples “Federico II”, Italy with swelling in the left suborbital region following a physical fight that had occurred 3 weeks before. Our patient reported that, during the attack, a pen had been used as a weapon to hurt him in the head and, despite previous surgical treatment, he complained of localized pain in the left suborbital region. He had no history of diabetes, no indications of immunosuppression and no allergies. He had no fever and reported neither nasal obstruction/discharge nor nosebleeds. An initial clinical examination revealed no abnormalities in his eyeballs or eye movement. Moderate edema and ecchymosis with bruising of his left lower eyelid were observed, with skin sutures at the orbital frame (Fig. 1). A neurological examination did not find any abnormalities. Palpation of the orbital contour revealed no fractures, and an ophthalmological evaluation showed no evidence of diplopia. Our patient had not received any recent dental care. An intraoral examination reported neither dental fractures nor mucosal lesions. Abnormalities on a complete blood cell count and serum biochemical profile only included a mild neutrophil elevation (13,000) and 3000 lymphocytes. No culture was taken because of the lack of nasal drainage. A computed tomography (CT) scan was the first line of investigation, and it revealed fractures in the left orbital floor and lateral wall of the maxillary sinus. Periorbital tissue herniation without muscular entrapment and left maxillary hemosinus were observed. A hypodense soft tissue mass (a foreign body) was lodged in the left orbital floor, which extended to the median wall of the maxillary sinus and penetrated the left lower turbinate. No zygomatic or upper jaw fractures were noted (Fig. 2). Surgical exploration of the foreign body was planned. Our patient was prescribed a preoperative antibiotic therapy (oral levofloxacin 500 mg four times a day) and the same dosage was given for 7 days post-surgery.

**Fig. 3:**
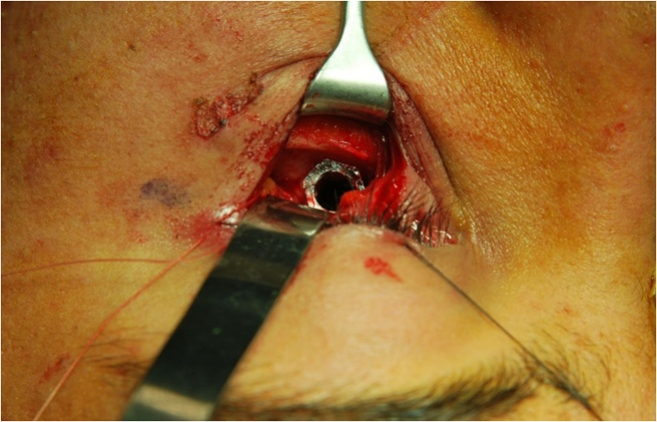
Intraoperative view of the surgical site. A foreign body is identified as a plastic pen cap measuring 0.5 cm in diameter

**Fig. 4:**
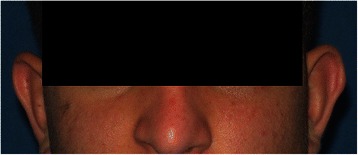
Soft tissue healing 3 months after surgery

**Fig. 5:**
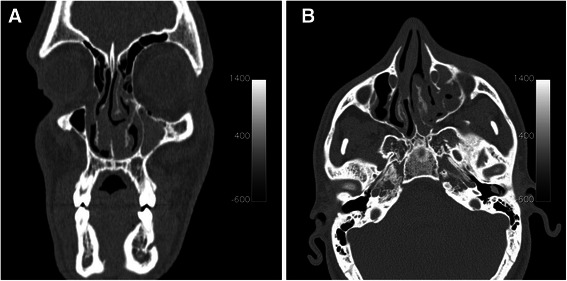
**a–b** Computed tomography image obtained 3 months after surgery. The reconstructed orbital floor and resolution of the left hemosinus are observed

Under general anesthesia, the orbital floor was exposed through the lower eyelid, and the foreign body was identified to be a plastic pen cap measuring approximately 0.5 cm in diameter (Fig. 3). The fractured portion of the orbital floor was slightly enlarged by piezosurgery to allow visualization of the plastic pen cap while preserving the bone at the site [7]. The pen cap was retrieved along the path of insertion without any hemorrhage, and following its removal, the residual bone wall was carefully probed to evaluate the bony defect. The foreign body, which was approximately 4.5 cm in length, had extended to the median wall of the left maxillary sinus and the lower turbinate, as observed on a preoperative CT scan. No regenerative procedures were required. Vicryl™ (polyglactin 91, 3 %) sutures (Ethicon, Somerville, NJ, USA) were used to close the flap. Immediately after surgery, no ophthalmological abnormalities were observed. Our patient’s postoperative course was uneventful, with no complications and remarkable soft tissue healing (Fig. 4). He was discharged a week later. Clinical and radiological follow-up performed 1 year after surgery, showed no abnormalities, with complete resolution of the hemosinus and adequate nasal and orbital reconstruction (Fig. 5).

## Discussion

Pure orbital floor fractures account for approximately 67–84 % of all orbital trauma cases [1, 3]. Occasionally, history or clinical examination alone may be inadequate to raise the suspicion of a retained periorbital foreign body. Therefore, CT is important for the evaluation of periorbital injuries [2]. The clinical course and indications for removal of the periorbital foreign body depend on a variety of factors, such as its position (near vital structures), chemical structure (many metals and plastic materials may be inert, whereas wood is associated with a higher incidence of complications), potentially infectious nature, or clinical findings (displacement or damage to vital anatomical structures and ophthalmological evaluation findings) [4, 8]. In some periorbital trauma the foreign body is dislocated in the maxillary sinus; many authors suggested its removal to prevent future infections [9, 10]. However, infection is not a certain outcome since sinuses have been observed to be healthy despite the inclusion of foreign materials [11]. The sinus can remain asymptomatic for several months before an acute infection develops. The removal of the foreign body is considered necessary because of peculiarities of the topographic anatomy of the maxillary sinus. The close proximity of the external posterior wall to the pterygopalatine fossa lodging the main trunk of the second division of the trigeminal nerve (the maxillary nerve), maxillary artery, and venos plexus connected with the orbit and cavernous sinus of the dura mater may promote the spread of pathology from the maxillary sinus to this region [12]. The superior wall of the maxillary sinus separates it from the orbit. On the surface of the wall lies a canal (sometimes a semicanal), which opens into the maxillary cavity and contains the maxillary nerve and vessels. Therefore, pathological conditions of the sinus may affect this vascular-nerve bundle or spread into the orbit [12]. Also the trigemino-cardiac reflex (TCR) reported by some authors is considered an indication for the removal of the intraorbital foreign body [13, 14]*.* Ophthalmological findings include the presence of double vision (Hess–Lancaster evaluation), enophthalmos/hypoglobus, and conjunctival ecchymosis [15].

With regard to radiological evaluation, CT is ideal for evaluating fractures of the orbital walls and the integrity of the adjacent sinuses [2]. Three-dimensional reconstruction after image acquisition allows categorization of the size and shape of fracture, thus aiding in surgical treatment planning [2]. Periorbital foreign bodies can be distinctly observed on CT, which remains the most sensitive study and should be the first imaging modality in such cases [16, 17]. We initially suspected a periorbital surface injury in our patient, and we considered CT to verify our suspicion of an orbital floor fracture, not to evaluate the potential presence of a periorbital foreign body. We were surprised to observe that a sizeable periorbital foreign body such as a plastic pen cap could find its way into the sinus through a small entry site, without showing major clinical manifestations such as sinusitis or sinus infection. The previous surgical treatment (orbital frame sutures) did not consider radiological findings; therefore, it was inadequate. Generally, the indications for surgical removal of foreign bodies include neurological compromise, mechanical restriction of ocular movements, development of acute or chronic infection, or chronic suppurative reactions, such as those observed with copper foreign bodies [18]. Removal of foreign bodies located close to the apex is also generally discouraged, because the risk of collateral damage far outweighs the benefits [19]. In our patient, surgical removal was considered to prevent sinus and periorbital infections.

## Conclusions

In conclusion, the findings from this case imply that in patients with major or minor periocular or ocular surface injury, clinical investigations should rely less on history, which may be misleading, and more on imaging studies such as CT, which allow for accurate diagnosis and surgical planning for the resolution of a traumatic process secondary to a retained foreign body that would remain undetected on clinical examination alone.

## Consent

Written informed consent was obtained from the patient for publication of this case report and any accompanying images. A copy of the written consent is available for review by the Editor-in-Chief of this journal.
